# ^18^F- FDG PET/CT-derived parameters predict clinical stage and prognosis of esophageal cancer

**DOI:** 10.1186/s12880-019-0401-x

**Published:** 2020-01-22

**Authors:** Styliani Mantziari, Anastasia Pomoni, John O. Prior, Michael Winiker, Pierre Allemann, Nicolas Demartines, Markus Schäfer

**Affiliations:** 1 Department of Visceral Surgery, Lausanne University Hospital (CHUV), Rue du Bugnon 46, 1011 Lausanne, Switzerland; 2 Faculty of Biology and Medicine, University of Lausanne (UNIL), Lausanne, Switzerland; 3 Department of Nuclear Medicine and Molecular Imaging, Lausanne University Hospital (CHUV), Lausanne, Switzerland

**Keywords:** Esophageal cancer, TNM stage, Nuclear imaging, Metabolic imaging, ^18^FDG PET/CT

## Abstract

**Background:**

Although ^18^F- FDG PET/CT is validated in baseline workup of esophageal cancer to detect distant metastases, it remains underused in assessing local staging and biology of the primary tumor. This study aimed to evaluate the association between ^18^F- FDG PET/CT-derived parameters of esophageal cancer, and its clinico-pathological features and prognosis.

**Methods:**

All patients (*n* = 86) with esophageal adenocarcinoma or squamous cell cancer operated between 2005 and 2014 were analyzed. Linear regression was used to identify clinico-pathologic features of esophageal cancer associated with the tumor’s maximal Standardized Uptake Value (SUV_max_), Total Lesion Glycolysis (TLG) and Metabolic Tumor Volume (MTV). ROC curve analysis was performed to precise the optimal cutoff of each variable associated with a locally advanced (cT3/4) status, long-term survival and recurrence. Kaplan Meier curves and Cox regression were used for survival analyses.

**Results:**

High baseline SUV_max_ was associated with cT3/4 status and middle-third tumor location, TLG with a cT3/4 and cN+ status, whereas MTV only with active smoking. A cT3/4 status was significantly predicted by a SUV_max_ > 8.25 g/mL (*p* < 0.001), TLG > 41.7 (*p* < 0.001) and MTV > 10.70 cm^3^ (*p* < 0.01) whereas a SUV_max_ > 12.7 g/mL was associated with an early tumor recurrence and a poor disease-free survival (median 13 versus 56 months, *p* = 0.030), particularly in squamous cell cancer.

**Conclusions:**

Baseline ^18^F- FDG PET/CT has a high predictive value of preoperative cT stage, as its parameters SUV_max_, TLG and MTV can predict a locally advanced tumor with high accuracy. A SUV_max_ > 12.7 g/mL may herald early tumor recurrence and poor disease-free survival.

## Background

Esophageal cancer is associated with aggressive lymphatic spread, resulting often in locally advanced or metastatic disease upon diagnosis [1]. Metabolic imaging with 18F- Fluorodeoxyglucose Positron Emission Tomography/Computerized Tomography (^18^F- FDG -PET/CT) has been integrated into the preoperative workup of esophageal cancer for the detection of distant suspicions lesions [1–3], interval metastases [4] or assessment of response to neoadjuvant treatment [5]. Esophageal cancer workup should use three-modality staging with Computerized Tomography (CT), endoscopic ultrasound (EUS) and ^18^F- FDG PET/CT [2, 3], as failure to identify locally advanced tumors (cT3/4 or N+) may lead to omission of neoadjuvant treatment before surgery, compromising patient survival [2, 6]. To this day, ^18^F- FDG PET/CT is not primarily used for local staging of the primary tumor due to its poor spatial resolution. However, Malik et al. recently demonstrated a significant predictive value of Metabolic Tumor Volume (MTV) in differentiating early-stage (cT1/2) from locally advanced (cT3/4) lesions [7]. Obtaining accurate cTN staging information through ^18^F- FDG PET/CT may be of prime importance particularly in cases where EUS is unavailable or if the tumor is obstructive (up to 19% of patients) [7]. Furthermore, although the predictive value of ^18^F- FDG PET/CT derived parameters maximal standardized Uptake Value (SUV_max_), Total Lesion Glycolysis (TLG) and MTV on long-term survival of esophageal cancer has been extensively reported in the literature [8], the absence of universally accepted thresholds and the paucity of data for each histological type limit the predictive value for the individual patient.

The aim of our study was to assess the clinico-pathological correlations and staging value of ^18^F- FDG PET/CT derived parameters SUV_max_, TLG and MTV, as well as their predictive value in patient survival and tumor recurrence.

## Methods

All patients operated for esophageal adenocarcinoma or squamous cell cancer, with curative intent, from 2005 to 2014 in our tertiary referral center and a baseline ^18^F- FDG PET/CT in the preoperative workup were included in this study. Demographic, clinical and histological data were retrieved from our prospectively maintained database.

In all patients, routine preoperative staging was performed by esophagogastroduodenoscopy, EUS and thoraco-abdominal CT scan. Since 2005 ^18^F- FDG PET/CT was integrated in the baseline preoperative workup, according to current recommendations [2]. TNM stage was defined according to the 7th TNM classification [9]. Neoadjuvant treatment was administered for locally advanced lesions (cT3/4 and/or N+), with 5FU-platin or carboplatin-paclitaxel based chemotherapy and external beam radiation of 41–54 Gy. R0 resection was defined as the presence of tumor within 1 mm of resection margins. Postoperative follow-up included a thoraco-abdominal CT scan every 4 months for the first two postoperative years and further workup in cases of suspected recurrence [10]. Early recurrence was defined as any documented recurrence in the first 12 postoperative months. Follow-up data were last updated in November 2018, to assure a minimum follow-up of 4 years for all patients.

### Baseline ^18^F- FDG PET/CT and derived parameters

Since the beginning of this study, we introduced our own PET/quality control program used in several national and international PET studies [11] until our center participated to the quality control program by the European Association of Nuclear Medicine (EANM)- EARL as PET/CT Center of Excellence in October 2011, for which we have been accredited each year so far. For ^18^F- FDG PET/CT, patients fasted for at least 6 h before and blood glucose was measured before administration of the radiotracer and required to be < 8.5 mmol/L, otherwise the scan was rescheduled. Each patient received 5.5 MBq/kg until 08/2011 or 3.5 MBq/kg thereafter of ^18^F- FDG intravenously and remained in a calm and warm area for 1 h post injection. Thereafter, the patient was asked to void and subsequently was placed in the scanner. Images were acquired on PET/CT scanner (Discovery LS until 08/2011 and thereafter Discovery D690 TOF; GE Healthcare, Waukesha, WI) with scatter and point-spread function recovery corrections. The CT scan (140 kV, 80 mA pith 1.5 until 08/2011 and thereafter 120 keV, 80–200 AutomA/SmartmA, pitch 1.375) was used for attenuation correction. The CT scan was followed by a PET over the same body region (3–5 min/bed position acquired in 2-D mode until 08/2011 thereafter 1 min 30 s–2 min/bed position acquired in 3-D mode). Images were reconstructed using OSEM (2 iterations, 26 subsets) with a 5.4-mm FWHM post-filter and 3.91-mm FWHM loop filter until 08/2011 and thereafter using OSEM (3 iterations, 16 subsets) with 5-mm postfilter and PSF and TOF recovery corrections. Two nuclear medicine physicians (AP, JOP) closely reviewed the images using for analysis an Advantage Workstation (version 4.6, GE Healthcare, Waukesha, WI) using PET VCAR software to compute SUV_mean_, SUV_max_, TLG and MTV.

SUV_max_ was defined as the point of maximal radiotracer uptake value within the delineated tumor volume (g/mL). MTV represents the metabolically active volume of the main tumor (cm^3^), whereas TLG was computed as the product of MTV multiplied by the tumor’s SUV_mean_. In order to define the contouring margin of primary tumor, a volume of interest around the tumor was drawn carefully to incorporate the target lesion in transaxial, sagittal and coronal planes. For tumor delineation we used a 42% threshold, as it is the most commonly used in the literature [7, 12].

### Statistical analysis

Linear regression was performed to assess correlation between several clinicopathological variables and baseline SUV_max_, TLG and MTV. For each PET/CT derived parameter, a Receiver Operating Characteristic (ROC) curve analysis was performed to assess whether an optimal cutoff could be associated with locally advanced lesions (cT3/4), overall survival and early tumor recurrence. Overall and disease-free survival were analyzed with the Kaplan-Meier method and log-rank test as well as and a Cox regression analysis. Based on previously published differences in FDG uptake between adenocarcinoma and squamous cell cancer [3, 13], subgroup analyses were separately carried out for each histological type. Co-variates with a *p*-value< 0.2 on a univariate level were entered to a backward elimination process, allowing to build the final multivariate model with the lowest Akaike Information Criterion (AIC) value. Significance level was set at *p* < 0.05 and all tests were two-sided. Statistical analysis was performed with RStudio (version 3.2.3, RStudio Team 2015, Boston, USA) and SPSS (version 23.0, Chicago, USA).

## Results

From the 141 patients operated in the study period, 89 had a baseline ^18^F- FDG PET/CT in their workup (63%). Three of them were excluded from analysis because of histology other than adenocarcinoma or squamous cell; thus, the current series consists of 86 patients. Baseline patient characteristics are outlined in Table 1. Adenocarcinoma represented 53% and squamous cell histology 47% of all lesions; 94% of all tumors were FDG-avid in ^18^F- FDG PET/CT.

**Table 1:** Baseline demographics and preoperative workup of all patients

Variable	*N* = 86
Median age, years [range]	63 [38–82]
Male Gender (%)	66 (77)
Active smoking (%)	38 (44)
ASA class (%)	
1–2	61 (71)
3–4	25 (29)
Tumor location (%)	
Upper third	3 (4)
Middle third	27 (31)
Distal third	29 (34)
Gastroesophageal junction	27 (31)
Clinical T stage (cT)	
cT1–2	21 (24)
cT3–4	63 (73)
Missing data	2 (2)
Clinical N stage (cN)	
cN0	33 (38)
cN+	49 (57)
Missing data	4 (5)
Tumor histology	
Adenocarcinoma	46 (53)
Squamous cell carcinoma	40 (47)
Preoperative workup	
CT	86 (100)
EUS	78 (91)
EUS non-obstructive lesion	73 (85)
^18^F- FDG PET/CT	86 (100)
^18^F- FDG PET/CT -avid lesion	81 (94)
Neoadjuvant treatment	71 (82)
Operative approach	
Transthoracic (Lewis)	65 (76)
Three-field (McKeown)	19 (22)
Transhiatal	2 (2)

*ASA* American Society of Anesthesiology, *CT* Computerized tomography, *EUS* Endoscopic ultrasound, ^*18*^*F- FDG PET/CT* ^18^F-Fluorodeoxyglucose Positron Emission Tomography CT

### Baseline ^18^F- FDG PET/CT -derived parameters and initial tumor staging

#### SUV_max_ (maximal standardized uptake value)

Median baseline SUV_max_ was 12.1 g/mL (range 2.8–48.0) for all tumors. Middle third tumor location, advanced cT and cN stage as well as squamous cell histology were associated with higher SUV_max_ values on a univariate level, however only tumor location and cT stage remained significant on multiple regression (Table 2). cT3/4 tumors had an expected SUV_max_ 6.61 higher than a cT1–2 lesion (β coefficient 6.61, 95%CI 2.40, 10.81, *p* = 0.002), and middle third tumors an expected SUV_max_ 7.01 higher than GEJ lesions (β coefficient 7.01, 95%CI 0.71–13.32, *p* = 0.029). The multivariable model presented a good fit to the data (*R*^2^ = 0.2804, F-statistic 4.676 on 6 and 72 DF, *p* < 0.0001).

**Table 2:** Linear regression analysis for baseline SUV_max_

Variable	Unadjusted β coefficient	95%CI	*P*-value	Adjusted β coefficient	95%CI	*P*-value
cT stage						
cT1–2	Ref			Ref		
cT3–4	7.76	3.64,11.87	< 0.001	6.61	2.40,10.81	0.002
cN stage						
cN0	Ref			Ref		
cN+	4.12	0.29,7.95	0.038	3.28	−0.49,7.05	0.087
Tumor location						
GEJ	Ref			Ref		
Distal third	1.41	−2.99,5.80	0.53	1.59	−3.10,6.29	0.50
Middle third	6.27	1.80,10.75	0.007	7.01	0.71,13.32	0.029
Superior third	−1.42	−11.26,8.42	0.78	−1.47	−12.05,9.11	0.78
Histology						
Adenocarcinoma	Ref					
Squamous cell	3.84	0.23,7.45	0.040	−0.630	−5.84,4.58	0.81

*SUV*_*max*_ Maximal standardized uptake value, *GEJ* Gastroesophageal junction, *95%CI* 95% confidence intervals, *Ref* Reference category (β coefficient = 0)

**Fig. 1:**
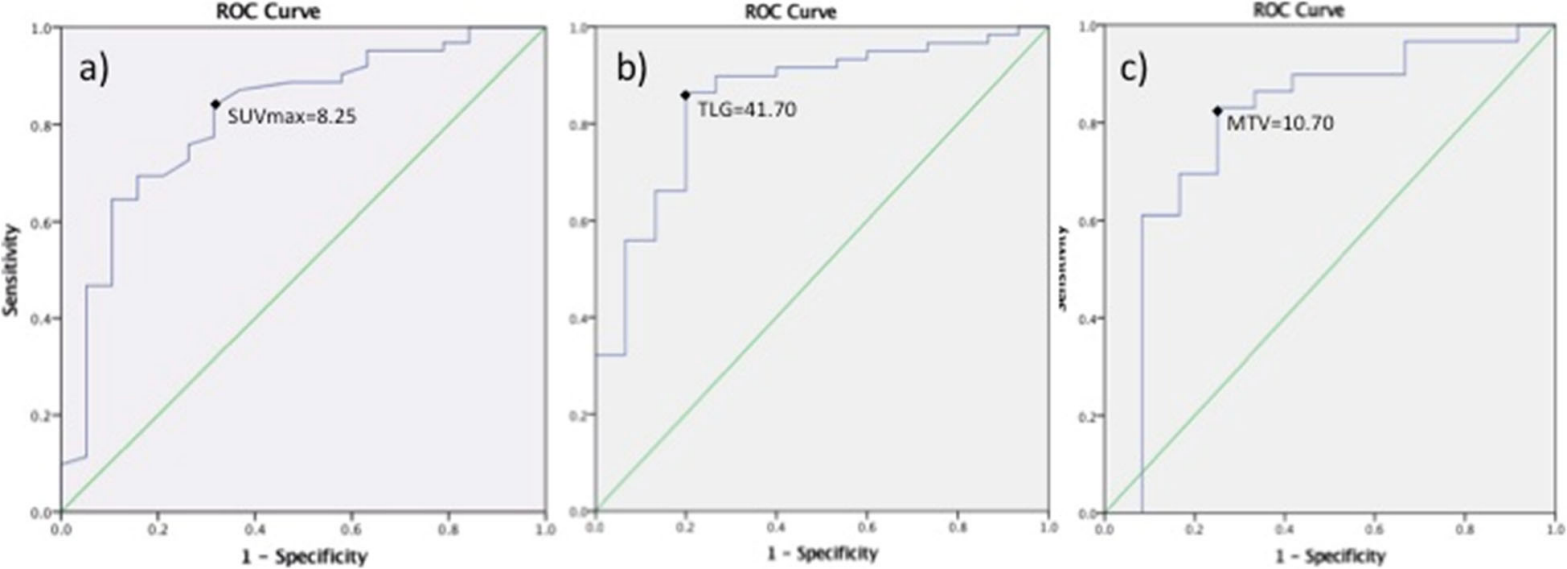
ROC curve analyses for the predictive value of ^18^FDG-PET/CT derived parameters in relation to a cT3/4 status. All three parameters predicted significantly cT3–4 status of the primary tumor. **a** SUV_max_ > 8.25 g/mL (sensitivity 83.9%, specificity 68.4%, *p* < 0.001), **b** TLG > 41.7 g (sensitivity 86.4%, specificity 80%, *p* < 0.001), **c** MTV > 10.7 cm^3^ (sensitivity 83.1%, specificity 75%, *p* = 0.01)

Baseline SUV_max_ presented a good prognostic value of a cT3/4 status in ROC curve analysis (Fig. 1a). A SUV_max_ > 8.25 g/mL predicted a cT3/4 lesion with a sensitivity of 84% and a specificity of 68%. Overall accuracy as indicated by the area under the curve (AUC) was 82% (AUC = 0.816, 95%CI = 0.704–0.928, *p* < 0.001).

#### TLG (Total lesion glycolysis)

Median TLG for all tumors was 122.1 (range 1–1179). Simple linear regression revealed higher TLG values for cT3/4 and cN+ tumors, and both co-variates remained significant in multivariate analysis; expected TLG for cT3/4 tumors was 162.9 higher than cT1/2 tumors (β coefficient 162.95, 95% CI 31.39–294.51, *p* = 0.016), and cN+ had a TLG 145.83 higher than cN0 lesions (β coefficient 145.83, 95% 34.47–256.19, *p* = 0.010). (Table 3) The model presented a good fit to the data (*R*^2^ = 0.1852, F-statistic 7.841 on 2 and 69DF, *p* < 0.001).

**Table 3:** Linear regression analysis for baseline TLG

Variable	Unadjusted β coefficient	95%CI	*P*-value	Adjusted β coefficient	95%CI	*P*-value
cT stage						
cT1–2	Ref			Ref		
cT3–4	198.42	61.42,335.43	0.006	162.95	31.39,294.51	0.016
cN stage						
cN0	Ref			Ref		
cN+	168.77	58.05,279.48	0.004	145.83	35.47,256.19	0.010

*TLG* Total lesion glycolysis, *95%CI* 95% confidence intervals, *Ref* Reference category (β coefficient = 0)

ROC curve analysis identified a TLG > 41.7 g as the optimal cutoff to detect a cT3/4 lesion, with a sensitivity of 86%, a specificity of 80% and an overall accuracy of 85% (AUC 0.852, 95%CI 0.744–0.960, *p* < 0.001) (Fig. 1b).

#### MTV (metabolic tumor volume)

Median MTV for all FDG-avid tumors was 22.7 cm^3^ (range 1–519). Univariate analysis identified only active smoking being associated with higher baseline MTV (β coefficient 32.81, 95%CI 4.99–70.62, *p* = 0.093) and thus, no multivariable analysis was possible for this parameter.

In ROC curve analysis a baseline MTV > 10.70 cm^3^ was identified as the optimal cutoff to predict cT3/4 status (sensitivity 83.1%; specificity 75%, AUC 0.799, 95%CI 0.640–0.959, *p* = 0.01) (Fig. 1c).

### Prognostic value of ^18^F- FDG PET/CT -derived parameters for recurrence and patient survival

**Fig. 2:**
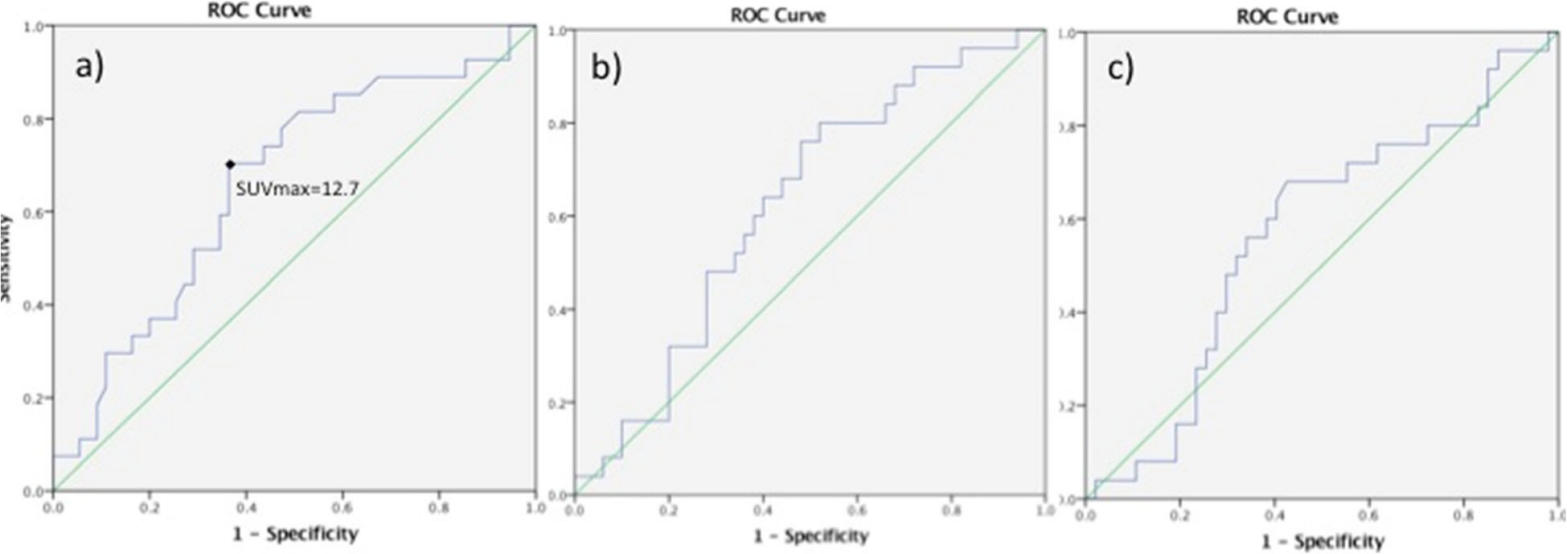
ROC curve analyses for ^18^FDG-PET/CT derived parameters as predictors of early tumor recurrence. **a** A SUV_max_ > 12.7 g/mL was identified as the optimal threshold for early recurrence, with a sensitivity of 70.4%, specificity 63.6% (AUC 0.660, *p* = 0.019). No optimal cutoff was defined for **b** TLG (AUC 0.624, 95% CI 0.495–0.753, *p* = 0.081) or **c** MTV (AUC 0.570, 95%CI 0.431–0.709, *p* = 0.332)

**Fig. 3:**
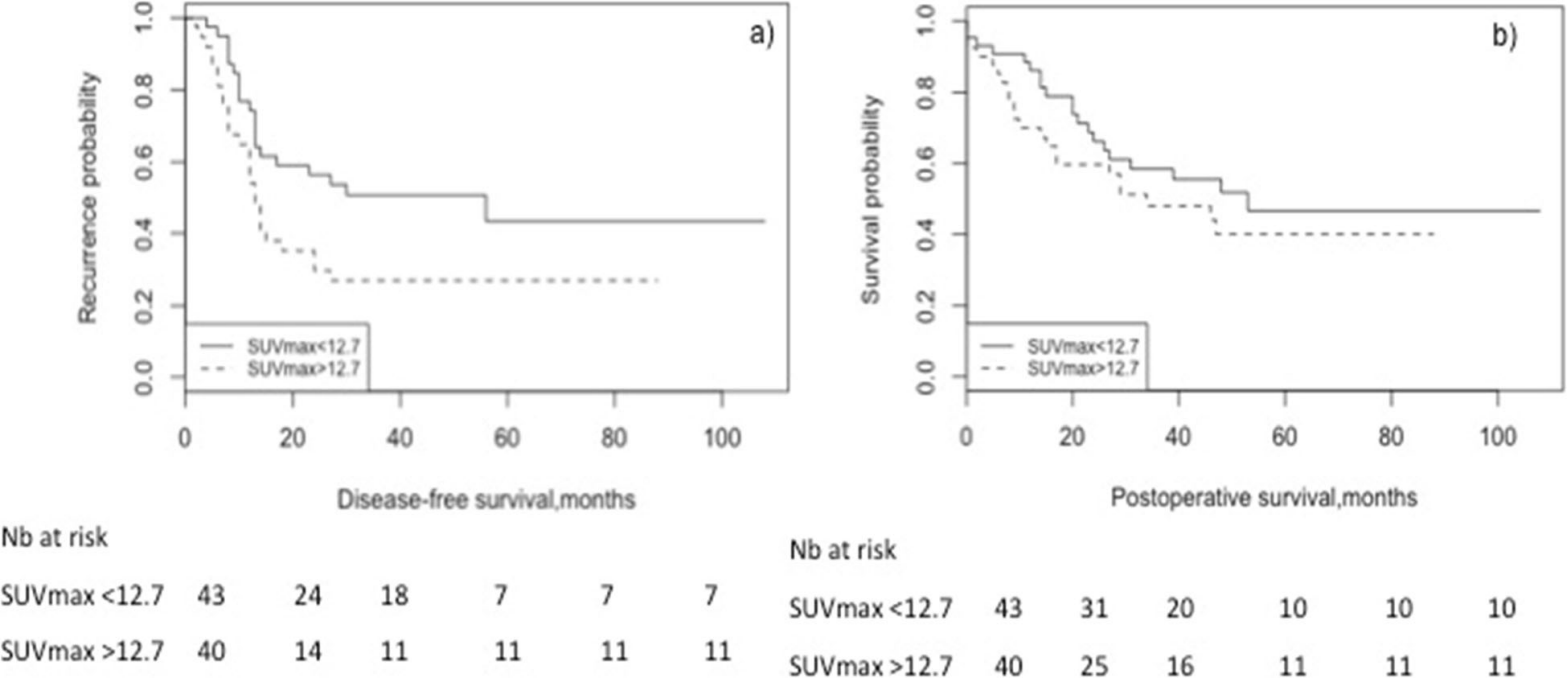
Baseline SUV_max_ as a predictor of disease-free and overall postoperative survival. **a** SUV_max_ > 12.7 g/mL was a significant predictor of poor disease-free survival (median DFS 13 versus 56 months, *p* = 0.030), **b** but not of overall survival

Among the three parameters studied, SUV_max_ at baseline was the only one with a significant predictive value for early tumor recurrence, within the 1st postoperative year (Fig. 2). A SUV_max_ > 12.7 g/mL predicted early recurrence with 70.4% sensitivity and 64.6% specificity (AUC 0.660, 95% CI 0.535–0.785, *p* = 0.019) (Fig. 2a). Patients with a SUV_max_ ≤ 12.7 g/mL at baseline had a median disease-free survival (DFS) of 56 months (95%CI 7.7–104), versus 13 (95%CI 10.4–15.7) for those with a SUV_max_ > 12.7 g/mL (*p* = 0.030) (Fig. 3). Cox regression confirmed SUV_max_ > 12.7 g/mL as an independent predictor of DFS (HR 2.54, 95%CI 1.26, 5.09, *p* = 0.009), along with preoperative active smoking and pT3/4 status (Table 4).

**Table 4:** Cox regression analysis for disease-free survival (DFS)

Variable	Unadjusted HR	95%CI	*P*-value	Adjusted HR	95%CI	*P*-value
All patients						
Active smoking	2.22	1.23,4.01	0.008	2.22	1.15,4.27	0.017
SUVmax> 12.7 g/mL	2.10	1.16,3.81	0.014	2.54	1.26,5.09	0.009
pT stage						
pT0	Ref			Ref		
pT1	0.30	0.06,1.45	0.135	0.59	0.11,3.04	0.532
pT2	1.32	0.49,3.57	0.577	1.55	0.56,4.28	0.393
pT3	2.78	1.18,6.55	0.019	3.31	1.37,8.00	0.008
pT4	11.93	2.25,63.29	0.004	7.24	1.34,39.22	0.022
Resection margins						
R0	Ref					
R+	2.47	1.04,5.89	0.041	2.09	0.77,5.68	0.147
Adenocarcinoma						
Active smoking	2.34	1.04–5.28	0.039	2.54	1.05–6.14	0.039
SUVmax> 12.7 g/mL	1.87	0.85–4.11	0.119	1.54	0.65–3.62	0.323
pN stage						
pN0	Ref			Ref		
pN1	5.76	2.06–16.09	< 0.001	4.04	1.29–12.58	0.016
pN2	5.24	1.78–15.49	0.003	3.88	1.25–12.02	0.019
pN3	9.31	1.82–47.49	0.007	8.67	1.50–50.07	0.016
Resection margins						
R0	Ref					
R+	4.37	1.44–13.25	0.009	2.78	0.78–9.94	0.115
Squamous cell cancer						
cT stage						
cT1–2	Ref			Ref		
cT3–4	2.74	0.92–8.10	0.069	0.96	0.19–4.78	0.961
SUVmax> 12.7 g/mL	1.82	0.76–4.36	0.176	5.06	1.44–17.71	0.011
pT stage						
pT0	Ref			Ref		
pT1	0.84	0.16–4.33	0.833	1.23	0.21–7.17	0.816
pT2	1.95	0.56–6.76	0.290	5.67	1.34–24.01	0.018
pT3	5.97	1.95–18.29	0.002	5.05	1.10–23.16	0.037
pT4	11.75	1.17–118.29	0.037	10.67	0.93–122.39	0.057
pN stage						
pN0	Ref			Ref		
pN1	0.22	0.03–1.67	0.144	0.09	0.01–0.92	0.042
pN2	4.51	1.42–14.31	0.010	4.71	1.00–22.14	0.049
pN3	4.39	0.95–20.32	0.058	6.72	0.90–49.88	0.062

*HR* Hazard ratio, *95%CI* 95% confidence intervals, *Ref* Reference category, *SUV*_*max*_ Maximal standardized uptake value

**Fig. 4:**
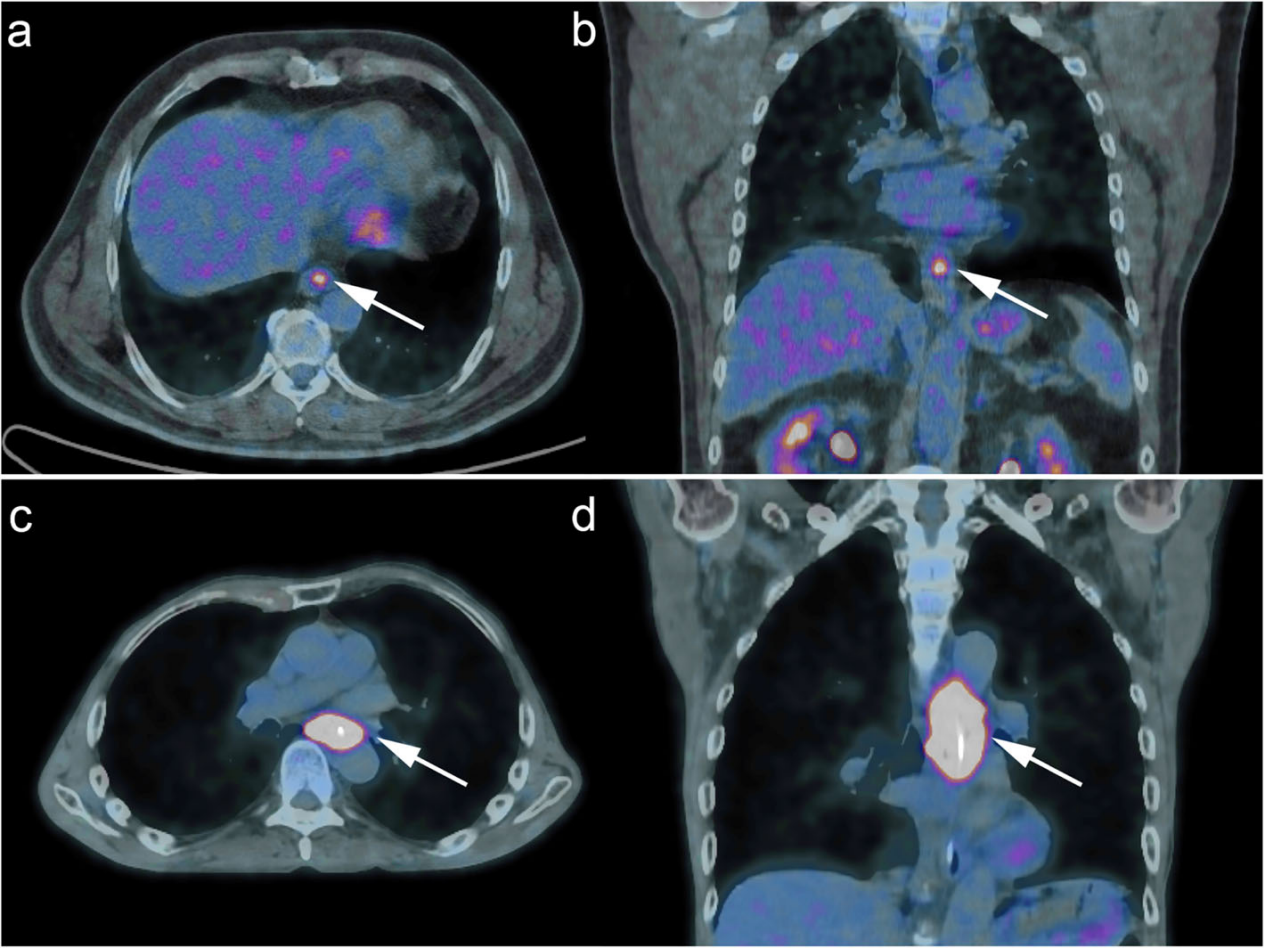
Axial and coronal ^18−^FDG PET/CT fusion images of two distinct esophageal malignancies with different metabolic profiles. Illustration of the distinct metabolic profile a cT1N0 adenocarcinoma of the gastroesophageal junction (Panels **a** and **b**, SUVmax = 5.5, TLG = 5) versus a cT3N+ squamous cell middle third lesion (Panels **c** and **d**, SUVmax = 12.8, TLG = 311.5). The white arrows show the primary tumor hypercaptation in each image

When the two histological subtypes were analyzed separately, there was no significant association of SUV_max_ with DFS for adenocarcinoma. For squamous cell carcinoma, a baseline SUV_max_ > 12.7 g/mL along with pT and pN stage independently predicted worse DFS (Table 4). The distinct metabolic profile of the two histological types is illustrated in Fig. 4.

No association was found between baseline SUV_max_ and overall survival (OS), neither on the Kaplan-Meier (Fig. 3) nor the Cox regression analysis. In the latter, only active smoking (HR 2.30, 95% CI 1.15–4.62, *p* = 0.019) and pT4 stage (HR 21.42, 95%CI 5.00–91.72, *p* < 0.0001) independently predicted OS. None of the variables remained independent predictors of OS in adenocarcinoma or squamous cell subtypes.

Median follow-up of all patients, calculated with the reverse Kaplan-Meier method, was 50 months (95%CI 45.33–54.66).

## Discussion

In this study, higher baseline SUV_max_ of esophageal cancer was significantly related to a middle-third tumor location and a cT3/4 stage, whereas higher TLG was related to cT3/4 and cN+ stage. Baseline SUV_max_ > 8.25 g/mL, TLG > 41.7 and MTV > 10.7 cm^3^ were associated with cT/4 stage, whereas SUV_max_ ≥ 12.7 g/mL predicted early recurrence and poor disease-free survival.

Currently, ^18^F- FDG PET/CT is mostly used for the detection of distant metastases as it can identify suspicious lesions as small as 1 cm [2, 14, 15]. Walker et al. reported ^18^F- FDG PET/CT -detected distant lesions precluding curative treatment in 21% of patients [15], even though the specificity of an FDG ‘hot spot’ remains low [16]. Until recently, limited spatial resolution of PET for esophageal wall layers and adjacent structures had restrained this modality as a detector of distant metastases [15]. Recent data, however, reinforce the role of ^18^F- FDG PET/CT in better defining cTNM stage and the tumor’s biology, the latter being FDG-avid in 84–92% of cases especially if it infiltrates the submucosa [7, 15, 17].

It is generally admitted that all ^18^F- FDG PET/CT-derived parameters are higher in advanced and aggressive tumors, however no universally accepted cutoffs exist to this day, limiting their cTNM staging value in the individual patient. Malik et al. suggested SUV_max_ > 4.1 (sensitivity 85%, and specificity 48%) and MTV > 23.4 cm^3^ (sensitivity 64%, specificity 67%) as optimal thresholds to distinguish cT1/2 from cT3/4 lesions [7]. In our study, SUV_max_ and TLG presented a higher predictive value than MTV in preoperative staging; ROC curve analysis yielded as significant cutoff values to predict preoperative cT3/4 status with high accuracy a SUV_max_ > 8.25 g/mL, TLG > 41.7 and MTV > 10.70 cm^3^. This discrepancy may be explained by the predominance of adenocarcinoma in the Malik study (75% of patients, versus 53% in the present study); adenocarcinoma has being described as less FDG-avid with lower SUV_max_ values compared to squamous cell cancer, probably in relation to increased expression of the HK-II biomarker [3, 13]. However, as in our study tumor histology was not independently associated with SUV_max_ (Table 2), no separate cutoffs of SUV_max_ were justified for each type.

One might argue that EUS is sufficient to identify locally advanced lesions (cT3/4 or N+) and thus to direct the patient to neoadjuvant treatment before surgery. However, previous data from our institution suggest a rather low rate of accurate usT (51%) and usN (72%) staging, with the highest rates of understaging among active smokers [18]. Indeed, a three-modality workup strategy (^18^F- FDG PET/CT, CT and EUS) offers the highest probability (84%) to correctly select patients for surgery, *a fortiori* when ^18^F- FDG PET/CT is the first exam performed [3]. Complementary use of these exams could improve staging accuracy, directing patients with locally advanced lesions to neoadjuvant treatment and avoiding its unnecessary toxicity for early-stage tumors.

Although several studies have reported poor long-term prognosis associated with high baseline SUV_max_ its prognostic value for the individual patient remains limited, as great variability is seen in the suggested cutoffs (ranging from 3 to 9 g/mL) [16, 19–22]. This variability might be linked to patient-related factors (e.g. cardiac output, tumor histology) but also to the PET/CT configuration and interobserver variability. To overcome these limitations, tumor-to-blood Standard Uptake Ratio (SUR) has been recently published as a promising predictor of survival [23, 24]. Hofheinz et al. [23] suggest the superiority of baseline SUR over SUV_max_ to predict overall survival in squamous cell cancer patients treated with definitive chemoradiation, whereas Bütof et al. [24] propose the restaging SUR value along with baseline MTV as a reliable survival predictor. In a recent meta-analysis, Han et al. reinforce the prognostic value of MTV and TLG for overall survival, although no specific cutoff value is suggested and both histological types are jointly taken into account [8]. In the present study none of the ^18^F- FDG PET/CT derived parameters demonstrated significant association with overall survival.

The added value of our study lies in the identification of SUV_max_ > 12.7 g/mL as an independent predictor of early tumor recurrence, within the 1st postoperative year. This result is of major clinical importance, as it might help early identification of patients with resectable esophageal cancer, who may not benefit from surgical resection as their risk of short-term recurrence is significantly increased. Indeed, we observed a significantly shorter DFS for squamous cell tumors with a baseline SUV_max_ > 12.7 g/mL; as definitive chemoradiation is a valid treatment option for this histological type, the above threshold may provide valuable prognostic information for the individual patient and guide accordingly therapeutic management. Schreurs et al. previously reported worse DFS for patients with baseline SUV_max_ > 3.67 g/mL, although no correlation with overall survival was found in that study either [3]. Markers of aggressive biology (GLUT-1, p53, Ki-67, HK-II) were studied in relation to SUV_max_, although no clear immunohistochemical profile was found for high-FDG uptake tumors compared to the others [3]. Thus, even though it is generally admitted that high baseline SUV_max_ may herald tumor aggressiveness and early recurrence, the underlying mechanism remains poorly understood. Our team previously reported active smoking as an independent predictor of early recurrence after esophagectomy [25], which is being confirmed in the present analysis along with a baseline SUV_max_ > 12.7 g/mL, and may express pathologic DNA-methylation patterns and tumor proliferation genes [26].

This study has some limitations that need to be addressed. Retrospective analysis has an inherent drawback in data completeness, even though our institutional database is maintained prospectively, with a stringent follow-up of all patients. The use of two different PET/CT scanners over the years might have introduce some bias in SUV_max_ measurements especially in the smaller-volume lesions, because of a better resolution recovery in the newer PET/CT scanner (from September 2011 and thereafter). However, both scanners measured similar SUV for lesions above 1.2 cm diameter (about 4 cm^3^). Since the mean MTV in our series was well above this threshold (48 cm^3^ ± 83 cm^3^), it can be estimated that this scanner change had little effect on the results. Moreover, we compared the SUV_max_ across both scanners in lesions with a MTV ≥30 cm^3^ (where SUV_max_ would be similar with both scanners) vs. lesions < 30 cm^3^, where the new scanner could measure SUV_max_ better. No differences were observed across scanners in the large lesions (MTV ≥ 30 cm^3^: SUV_max_ 20.6 ± 7.8 g/mL for the new scanner vs. 17.3 ± 12.0 g/mL for the previous one, *p* = 0.36). This was also the case for the smaller lesions (MTV < 30 cm^3^: SUV_max_ 9.9 ± 4.9 g/mL for the new scanner vs. 11.9 ± 4.4 g/mL for the previous one, *p* = 0.17). Results of this comparative analysis between the two CT scans are provided as supplementary material, and enhance our belief that the scanner change could not influence significantly our results.

Although there was practically no heterogeneity in ^18^F- FDG PET/CT results over the years, inaccuracies in the preoperative workup may have occurred for both T and N staging especially with EUS, confounding associations with ^18^F- FDG PET/CT parameters. As mentioned above, histological type and patient-related factors may influence ^18^F- FDG PET/CT-derived parameters and limit the use of universally accepted cutoffs. In the present study, due to the small number of patients per histological type our subgroup analyses can be considered as exploratory, needing validation in larger cohorts. Prospective validation of the suggested cutoffs should take into account the specificities of histological types studied.

## Conclusions

^18^F- FDG PET/CT derived parameters SUV_max_ > 8.25 g/mL, TLG > 41.7 g and MTV > 10.70 cm^3^ were significantly associated with locally advanced cT3/4 stage and a baseline SUV_max_ > 12.7 g/mL with early tumor recurrence and poor disease-free survival, particularly for squamous cell cancer. These findings add to the existing knowledge of ^18^F- FDG PET/CT’s value in esophageal cancer management and reinforce its predictive value in terms of tumor recurrence and survival. A prospective study is currently running in our institution to correlate these values with high-resolution CT and MR imaging characteristics of the primary tumor.

## Supplementary information

**Additional file 1.** Comparison of the SUVmax values for large (MTV>=30cm^3^) and small (MTV<30cm^3^) tumors, for the two CT scanners used in the study.

## Data Availability

The data that support the findings of this study are available from authors upon request and with permission of the Lausanne University Ethics Committee.

## Abbreviations

^18^F- FDG PET/CT: 18F- Fluorodeoxyglucose Positron Emission Tomography/Computerized Tomography
5-FU: 5 Fluoro-Uracil
95%CI: 95% Confidence Intervals
AIC: Akaike Information Criterion
ASA: American Society of Anesthesiologists
AUC: Area under the curve
CT: Computerized Tomography
DFS: Disease-free survival
EANM: European Association of Nuclear Medicine
EARL: EANM Research Limited
EUS: Endoscopic ultrasonography
GEJ: Gastroesophageal junction
HR: Hazard ratio
MTV: Metabolic Tumor Volume
OS: Overall survival
ROC: Receiver Operator Characteristic
SUR: Standard Uptake Ratio
SUVmax: Maximal Standardized Uptake Value
TLG: Total Lesion Glycolysis

## References

[1] Pennathur A, Gibson MK, Jobe BA (2016). Oesophageal carcinoma. Lancet.

[2] Lordick F, Mariette C, Haustermans K (2016). Oesophageal cancer: ESMO clinical practice guidelines for diagnosis, treatment and follow-up. Ann Oncol.

[3] Schreurs LMA, Janssens ACJW, Groen H (2016). Value of EUS in determining curative resectability in reference to CT and FDG-PET: the optimal sequence in preoperative staging of esophageal cancer?. Ann Surg Oncol.

[4] Kroese TE, Goense L, van Hillegersberg R, et al. Detection of distant interval metastases after neoadjuvant therapy for esophageal cancer with 18F-FDG PET(/CT): a systematic review and meta-analysis. Dis Esophagus. 2018;31(12). 10.1093/dote/doy055. 10.1093/dote/doy055 29917073

[5] Lordick F, Ott K, Krause BJ (2007). PET to assess early metabolic response and to guide treatment of adenocarcinoma of the oesophagogastric junction: the MUNICON phase II trial. Lancet Oncol.

[6] Pasquali S, Yim G, Vohra RS (2017). Survival after neoadjuvant and adjuvant treatments compared to surgery alone for resectable esophageal carcinoma: a network meta-analysis. Ann Surg.

[7] Malik V, Johnston C, O’Toole D (2017). Metabolic tumor volume provides complementary prognostic information to EUS staging in esophageal and junctional cancer. Dis Esophagus.

[8] Han S, Kim YJ, Woo S (2018). Prognostic value of volumetric parameters of pretreatment 18F-FDG PET/CT in esophageal cancer: a systematic review and meta-analysis. Clin Nucl Med.

[9] Sobin LH, Gospodarowicz MK, Wittekind C. Oesophagus including oesophagogastric junction malignant tumors. In: TNM UICC classification of malignant tumours. 7th ed: Wiley-Blackwell Editions; 2011. p. 66–72.

[10] Allemann P, Mantziari S, Wagner D (2016). Curative treatment for esophageal cancer. Results of a multidisciplinary consensus; traitement curatif du cancer de l’oesophage: consensus multidisciplinaire multicentrique. Rev Med Suisse.

[11] Gnesin S, Deshayes E, Camus F (2017). Quantification and monitoring of PET/CT data in multicentre trials: the Swiss SAKK 56/07 trial experience. Médecine Nucléaire.

[12] Xu W, Yu S, Ma Y (2017). No Effect of different segmentation algorithms on metabolic tumor volume measured on 18F-FDG PET/CT of cervical primary squamous cell carcinoma. Nucl Med Commun.

[13] Imperiale A, Cimarelli S, Brigand C (2011). Does the association of 18F-FDG uptake intensity and lesion topography reveal histological phenotype and tumor differentiation in esophageal cancer?. Hell J Nucl Med.

[14] van Vliet EP, Heijenbrok-Kal MH, Hunink MG (2008). Staging investigations for oesophageal cancer: a meta-analysis. Br J Cancer.

[15] Walker A, Spier B, Perlman S (2011). Integrated PET/CT fusion imaging and endoscopic ultrasound in the pre-operative staging and evaluation of esophageal cancer. Mol Imaging Biol.

[16] Malik V, Johnston C, Donohoe C (2012). (18)F-FDG PET-detected synchronous primary neoplasms in the staging of esophageal cancer: incidence, cost, and impact on management. Clin Nucl Med.

[17] Kato H, Nakajima M, Sohda M (2009). The clinical application of 18F-fluorodeoxyglucose positron emission tomography to predict survival in patients with operable esophageal cancer. Cancer.

[18] Winiker M, Mantziari S, Figueiredo SG (2018). Accuracy of preoperative staging for a priori resectable esophageal cancer. Dis Esophagus.

[19] Omloo JMT, Sloof GW, Boellaard R (2008). Importance of fluorodeoxyglucose-positron emission tomography (FDG-PET) and endoscopic ultrasonography parameters in predicting survival following surgery for esophageal cancer. Endoscopy.

[20] Cerfolio RJ, Bryant AS (2006). Maximum standardized uptake values on positron emission tomography of esophageal cancer predicts stage, tumor biology, and survival. Ann Thorac Surg.

[21] Rizk N, Downey RJ, Akhurst T (2006). Preoperative 18[F]-fluorodeoxyglucose positron emission tomography standardized uptake values predict survival after esophageal adenocarcinoma resection. Ann Thorac Surg.

[22] Hatt M, Visvikis D, Albarghach NM (2011). Prognostic value of 18F-FDG PET image-based parameters in oesophageal cancer and impact of tumour delineation methodology. Eur J Nucl Med Mol Imaging.

[23] Hofheinz F, Li Y, Steffen IG (2019). Confirmation of the prognostic value of pretherapeutic tumor SUR and MTV in patients with esophageal squamous cell carcinoma. Eur J Nucl Med Mol Imaging.

[24] Bütof Rebecca, Hofheinz Frank, Zöphel Klaus, Schmollack Julia, Jentsch Christina, Zschaeck Sebastian, Kotzerke Jörg, van den Hoff Jörg, Baumann Michael (2018). Prognostic Value of Standardized Uptake Ratio in Patients with Trimodality Treatment of Locally Advanced Esophageal Carcinoma. Journal of Nuclear Medicine.

[25] Mantziari S, Allemann P, Winiker M, Demartines N, Schäfer M (2018). Locoregional tumor extension and preoperative smoking are significant risk factors for early recurrence after esophagectomy for cancer. World J Surg.

[26] Kaz AM, Grady WM, Stachler MD, Bass AJ (2015). Genetic and epigenetic alterations in Barrett’s esophagus and esophageal adenocarcinoma. Gastroenterol Clin North Am.

