# Supplementary material for: 18F- FDG PET/CT-derived parameters predict clinical stage and prognosis of esophageal cancer
Source: BMC Med Imaging. 2020 Jan 22;20:7. doi: 10.1186/s12880-019-0401-x (PMC6977262; doi:10.1186/s12880-019-0401-x)
Supplement: Supplementary file 1 — Additional file 1. Comparison of the SUVmax values for large (MTV>=30cm3) and small (MTV<30cm3) tumors, for the two CT scanners used in the study. [file 12880_2019_401_MOESM1_ESM.docx]

Online appendix. Comparison of the SUVmax values for large (MTV>=30cm^3^) and small (MTV<30cm^3^) tumors, for the two CT scanners used in the study.

We specifically studied the potential effect of the newer CT scan in our sample, according to MTV above or below 30cm^3^ corresponding to the expected limit where partial volume effects would start to have a decrease on the measured SUVmax. We could not see any differences in the large MTV volumes (MTV≥30cm^3^: SUVmax 20.6±7.8 g/mL for the newer scanner vs. 17.3±12.0 g/mL for the older one, p=0.36). This was also the case for the smaller volumes MTV<30cm^3^ (SUVmax 9.9±4.9 g/mL for the newer scanner vs. 11.9±4.4 g/mL for the older one, p=0.17).
